# SMYD2 regulates lymph node metastasis derived from intrahepatic cholangiocellular carcinoma through CCR7-dependent chemotaxis

**DOI:** 10.1038/s41598-026-55155-y

**Published:** 2026-06-01

**Authors:** Masashi Nakamura, Shinya Hayami, Atsushi Miyamoto, Norihiko Suzaki, Tomohiro Yoshimura, Kensuke Nakamura, Yoshinobu Shigekawa, Atsushi Shimizu, Yuji Kitahata, Akihiro Takeuchi, Hideki Motobayashi, Kyohei Matsumoto, Shogo Ehata, Ryuji Hamamoto, Manabu Kawai

**Affiliations:** 1 Second Department of Surgery, School of Medicine, Wakayama Medical University, 811-1 Kimiidera, Wakayama, 641-8510 Japan; 2 Department of Pathology, School of Medicine, Wakayama Medical University, 811-1 Kimiidera, Wakayama, 641-8510 Japan; 3 Division of Medical AI Research and Development, National Cancer Center Research Institute, 5-1-1 Tsukiji, Chuo-ku, Tokyo, 104-0045 Japan

**Keywords:** SMYD2, Intrahepatic cholangiocellular carcinoma (ICC), Lymph node metastasis (LNM), NF-κB p65, CCR7, Cancer, Oncology

## Abstract

Intrahepatic cholangiocellular carcinoma (ICC) has poor prognosis, especially with lymph node (LN) metastasis (LNM). Lysine methyltransferase SET and MYND domain-containing 2 (SMYD2) has critical roles and is reportedly associated with poor prognosis in various cancers, but the role of SMYD2 in ICC is unclear. Here, we examined the role of SMYD2 in the progression of LNM of ICC. We immunohistochemically analyzed SMYD2 expression using resected specimens first in primary ICC and then in metastatic LNs derived from ICC. For functional analyses, we then performed cell proliferation assay following SMYD2 inhibition using two ICC cell lines such as HuCCT-1 and HuH28. To explore the detailed mechanisms of LNM, immunocytochemistry and western blotting related to epithelial-mesenchymal transition (EMT) were first performed. Moreover, we performed clinically immunohistochemical staining of C-C motif chemokine receptor 7 (CCR7) in metastatic LNs, immunofluorescence staining and western blotting for functional analyses to evaluate the relationship between SMYD2 and CCR7. Finally, p65 of nuclear factor-kappa B (NF-κB) and phospho-p65 (Ser536) status were checked as the regulator of CCR7. Protein expression levels of SMYD2 in the primary ICC lesion were not associated with any clinicopathological characteristics or prognosis. However, SMYD2 was expressed in metastatic LNs of all cases and LNM was strongly associated with poor disease-free and overall survival also as previously reported. This study therefore aimed to clarify the molecular-biological roles for LNM by SMYD2. SMYD2 knockdown significantly inhibited ICC cell proliferation. According to subsequent EMT-related research, SMYD2 knockdown did not induce morphological changes or cadherin switching. Next, chemotaxis assay revealed that SMYD2 knockdown reduced chemotaxis in HuCCT-1 from metastatic ascites but not in HuH28 from the primary lesion. CCR7 expression in metastatic LNs was immunologically confirmed in all cases and had a positive correlation with SMYD2 expression. Decreased expression of CCR7 was observed in SMYD2-suppressed HuCCT-1 cells by immunofluorescence staining and western blotting simultaneously with the decreasing phosphorylation of p65^S536^. SMYD2 could be demonstrated to express in all metastatic LNs derived from ICC and may regulate this metastatic mechanism through CCR7-dependent chemotaxis rather than EMT via the SMYD2-phospho-p65^S536^-CCR7 pathway.

**Supplementary Information:**

The online version contains supplementary material available at 10.1038/s41598-026-55155-y.

## Background

Intrahepatic cholangiocellular carcinoma (ICC) is the second most common liver primary cancer after hepatocellular carcinoma. In Japan, the percentage of ICC in primary liver cancer, now approximately 6.9%, has been gradually increasing. There were a number of previous reports that ICC has poor prognosis, especially with lymph node metastasis (LNM)^1–7^. Surgical treatment is the only curative therapy for ICC, but only 56.3% of patients with ICC can receive surgery as the initial treatment^1^. Most cases without surgical indications (including recurrent cases) are eligible for drug therapy. Combination therapy with gemcitabine plus cisplatin (GC) has long been accepted as the first-line regimen for biliary tract cancers including ICC^8^. The addition of S-1 to GC therapy has recently been shown to improve the prognoses of cases with unresectable biliary tract cancers compared with GC therapy, but median overall survival (OS) of this therapy remains at 13.5 months^9^. Moreover, combination therapies with immune checkpoint inhibitors such as durvalumab and pembrolizumab have been clinically applied and spread as treatment options^10,11^, but therapeutic effects also remain insufficient. The established agents (except immune checkpoint inhibitors) exert antitumor effects as cytotoxic chemotherapeutic agents, so there is a need for new agents focusing on novel mechanisms.

We have continued to research post-translational modifications in malignant tumors, especially focusing on methyltransferases and demethylases including SET and MYND domain-containing 2 (SMYD2, also named as Lysine N-Methyltransferase 3C: KMT3C)^12–24^. SMYD2 is the lysine methyltransferase that methylates either histones such as H3K36 and H3K4 or non-histone proteins such as p53^25^, retinoblastoma 1 (RB1)^12^, poly [ADP-ribose] polymerase 1 (PARP1)^26^, and Heat Shock Protein 90 Alpha Family Class B Member 1 (HSP90AB1)^27^, and this results in tumor progression and carcinogenesis. Suppression of SMYD2 has been shown to inhibit tumor progression. For example, RB1 methylation by SMYD2 induces to promote phosphorylation by cyclin dependent kinase 4 (CDK4), resulting in the separation of E2F from RB1, and isolated E2F accelerates cell cycle progression^12^. Overexpression of SMYD2 was reportedly associated with tumor progression and poor prognosis in various cancers including esophageal squamous cell carcinoma^28^, bladder cancer^12^, gastric cancer^29^, breast cancer^30^ and pediatric acute lymphoblastic leukemia^31^. However, there have been no reports on the role and association with prognosis in ICC by SMYD2.

LNM has been reported as one of the poorest prognostic factors in ICC^1–7^, but its molecular mechanisms remain poorly understood. SMYD2 was reportedly to be associated with LNM in several malignancies, including gastric cancer^29^ and colorectal cancer^32^. In colorectal cancer, for example, SMYD2 represses APC regulator of WNT signaling pathway 2 (APC2) expression by recruiting DNMT1 to its promoter, leading to activation of the Wnt/β-catenin pathway and subsequent induction of epithelial-mesenchymal transition (EMT), thereby promoting metastasis^32^. These findings suggest that SMYD2 may contribute to metastatic progression through context-dependent signaling pathways. However, it is unclear whether SMYD2 regulates LNM in ICC, and whether its effect involves EMT or alternative mechanisms. Here, we investigated the potential involvement of SMYD2 in LNM derived from ICC.

## Methods

### Patients

Between 2001 and 2015, 71 cases with ICC underwent primary hepatectomy at our hospital. We excluded three cases from the analysis due to perioperative death within 30 days after surgery. This study therefore focused on the 68 remaining cases.

### Immunohistochemistry

We randomly selected formalin-fixed paraffin-embedded tissue sections of primary ICC including corresponding background liver and metastatic LNs. Sections of hepatic hemangioma and non-metastatic LNs were prepared as a negative control for each primary ICC and metastatic LNs. Sections were deparaffinized in xylene and rehydrated in graded alcohol baths. Antigen retrieval was performed as follows: 0.25% trypsin-EDTA for 30 min at 37 ℃ for SMYD2 and antigen retrieval solution citrate pH6.0 (S2369, Dako, Glostrup, Denmark) for 10 min at 95 ℃ under high pressure for C-C motif chemokine receptor 7 (CCR7). Sections were then treated with 0.3% hydrogen peroxide in methanol for 30 min for SMYD2 or 10 min for CCR7 at room temperature to block endogenous peroxidase activity. Non-specific binding was blocked with the following reagents at room temperature: serum-free protein blocking reagent (X0909, Dako) for 15 min for SMYD2, and 3% bovine serum albumin in phosphate-buffered saline for 1 h for CCR7. The sections were then incubated with the following primary antibodies at room temperature: SMYD2 (sc-393827, Santa Cruz Biotechnology, Dallas, TX, 1:200 for liver sections and 1:100 for LN sections) for 30 min or CCR7 (25898-1-AP, Proteintech, Rosemont, IL, 1:200) for 1 h. The sections were washed with phosphate-buffered saline and incubated with the following reagents for 30 min at room temperature: anti-mouse Envision+System-HRP labelled polymer (K4001, Dako) for SMYD2, and anti-rabbit Envision+System-HRP labelled polymer (K4003, Dako) for CCR7. After washing with phosphate-buffered saline, the sections were reacted with Histofine DAB substrate kit (#425011, Nichirei Biosciences Inc., Tokyo, Japan) for 5 min and treated with Mayer’s hematoxylin solution (#131–09665, Fujifilm Wako Pure Chemical Corporation, Osaka, Japan) for 4 min as a nuclear counterstain. Slides were dehydrated through graded alcohol to xylene wash and mounted on coverslips.

Immunohistochemistry staining results were evaluated by the immunoreactive scoring (IRS) system^23,24,33^. The percentage of positive cells was graded (0: none, 1: 1%−25%, 2: 26–50%, 3: 51%−75%, 4: 76%−100%), the intensity of positive cells was scaled (0: negative, 1: weak, 2: moderate, 3: strong), and then the IRS was calculated by multiplying these scores. Scoring was performed individually by three investigators (M.N., S.H. and N.S.) blinded to the clinicopathological variables. Thorough discussion was conducted to reach consensus when the scoring differed between the investigators. IRS was finalized by the average of the scores of three investigators and one independent pathologist (S.E.).

### Cell lines and cultures

Two ICC cell lines, HuCCT-1 (CVCL_0324; derived from malignant ascites cells) and HuH28 (CVCL_2955; derived from the primary liver lesion), were purchased from RIKEN BioResource Research Center (Ibaraki, Japan) in September 2020. HuCCT-1 was cultured in RPMI-1640 medium, and HuH28 was cultured in MEM medium containing 10% fetal bovine serum (FBS). These cells were incubated at 37 ℃ in 5% CO_2_ conditions.

### SMYD2 knockdown by specific small interfering RNA (siRNA) transfection

The two siRNA oligonucleotide duplexes (Merck Sigma-Aldrich, Darmstadt, Germany) targeting SMYD2 (siSMYD2 #1 and #2) and one siRNA targeting for enhanced green fluorescent protein (EGFP), as a negative control, were customized and synthesized in the same way as previously reported^12^. The sequences of each siRNA are shown in Supplementary Table S1. These siRNAs were reverse-transfected (50 nM final concentration) into ICC cells using Lipofectamine™ RNAiMAX (#13778150, Thermo Fisher Scientific, Waltham, MA, US) in Opti-MEM™ (#31985-088, Thermo Fisher Scientific) according to the manufacturers’ protocol. ICC cells were incubated with siRNA for 48 h except the cell proliferation assay and examined for the assays below.

### Quantitative reverse-transcriptional polymerase chain reaction (qRT-PCR)

Specific primers for human glyceraldehyde-3-phosphate dehydrogenase (housekeeping gene) and SMYD2 were customized for qRT-PCR (Merck Sigma-Aldrich) in the same manner as previously reported^12^. The sequences of each primer are shown in Supplementary Table S2. Total RNA was extracted from ICC cells using RNeasy^®^ Plus Mini Kit (#74134, QIAGEN, Venlo, Netherlands), and complementary DNA was synthesized from 1 µg of total RNA using SuperScript^®^ III First-Strand Synthesis System (#18080051, Thermo Fisher Scientific). Then, 0.2 µl each of forward and reverse primers (100 µM), 10 µl of SsoAdvanced™ Universal SYBR^®^ Green Supermix (#1725271, Bio-Rad Laboratories, Hercules, CA) and 7.6 µl of RNase free water were added to 2 µl of complementary DNA solution, and qRT-PCR was performed using CFX96 touch^®^ (Bio-Rad Laboratories) according to the manufacturers’ protocol. Relative expression levels of messenger RNA were calculated with the 2-ΔΔCt method.

### Cell proliferation assay

Each siRNA was reverse-transfected (50 nM final concentration) into ICC cells in 96-well plate at a density of 1.0 × 10^4^ cells per well with Opti-MEM. After incubation for 72 h, Cell Counting Kit-8 (Dojindo, Kumamoto, Japan) was applied according to the manufacturers’ protocol, and cells were incubated for 2 h. After the incubation, absorbance at 450 nm was measured in a microplate reader (SH-9000, Corona Electric, Ibaraki, Japan).

### Immunocytochemistry

siRNA-transfected ICC cells were fixed with 10% neutral buffered formalin for 30 min and treated with 0.3% hydrogen peroxidase/phosphate-buffered saline to block endogenous peroxidase activity. After the treatment with serum-free protein blocking reagent (X0909, Dako) for 15 min, cells were incubated with anti-SMYD2 antibody (sc-393827, Santa Cruz Biotechnology, 1:200) for 30 min. Cells were then treated with anti-mouse Envision+System-HRP labelled polymer (K4001, Dako) for 30 min and reacted with Histofine DAB substrate kit (#425011, Nichirei) for 5 min. Finally, cells were treated with Mayer’s hematoxylin solution (#131–09665, Wako) for 4 min as a nuclear counterstain. All procedures were performed at room temperature.

### Western blotting

Cell lysates were prepared using CelLytic™ M (C3228, Sigma-Aldrich, St. Louis, MO) with phosphatase inhibitor cocktail (07575-51, NACALAI TESQUE, INC., Kyoto, Japan) and protease inhibitor cocktail (#635673, Takara Bio USA, Inc., San Jose, CA). Concentrations of cell lysates were measured using a BCA protein assay kit (T9300A, Takara Bio Inc., Shiga, Japan), and 15 µg total protein/lane was separated by standard sodium dodecyl sulfate-polyacrylamide gel electrophoresis (SDS-PAGE) using NuPAGE™ Bis-Tris Mini Protein Gels, 12%, 1.0 mm (NP0341, Thermo Fisher Scientific) under reducing conditions and blotted onto polyvinylidene difluoride membranes (#1704156, Bio-Rad Laboratories). Membranes were blocked with 5% skimmed milk-TBS-Tween for 1 h and cut along the lanes prior to the primary antibody reaction when necessary. Then membranes were incubated with the primary antibodies against the following proteins; SMYD2 (sc-393827, Santa Cruz Biotechnology, 1:200) and β-actin (sc-47778, Santa Cruz Biotechnology, 1:200) for 1 h at room temperature, epithelial cadherin (E-cadherin) (#3195, Cell Signaling Technology, Danvers, MA, 1:500), neural cadherin (N-cadherin) (#14215, Cell Signaling Technology, 1:500), CCR7 (25898-1-AP, Proteintech, 1:1000), p65 of nuclear factor-kappa B (NF-κB) (ab16502, Abcam, Cambridge, UK, 1:1000) and phospho-p65 (Ser536) (#3033, Cell Signaling Technology, 1:1000) overnight at 4℃.

After washing in TBS-Tween for 30 min, the membranes were incubated with secondary antibodies; anti-mouse IgG (#7076, Cell Signaling Technology, 1:1000) or anti-rabbit IgG, HRP-linked antibody (#7074, Cell Signaling Technology, 1:1000) for 1 h at room temperature. After washing in TBS-Tween for 30 min, the specific immunoreactive proteins were visualized by chemiluminescence (RPN2236, Cytiva, Marlborough, MA). The bands were quantified using ImageJ software (NIH, US), and band intensities were normalized to β-actin.

### Chemotaxis assay

Transwell plates with a polycarbonate membrane (8.0 μm pores) separating the upper and lower chambers (#3422, Corning, Corning, NY) were used. We added 200,000 siRNA-transfected cells in 200 µl FBS free medium to the upper chamber and added 600 µl 10% FBS containing medium to the lower chamber. After 20 h of incubation at 37 ℃ in 5% CO_2_ conditions, the inserts were gently wiped three times with a water-moistened cotton swab to remove detached or dead cells. Cells remaining on the upper chamber were then stained with cell stain solution (#11002, Cell Biolabs. Inc., San Diego, CA) for 10 min at room temperature. The inserts were gently washed three times in a beaker of water and allowed to air dry. Cells were then extracted with 200 µl of extraction solution (#11003, Cell Biolabs. Inc.) for 10 min at room temperature, and 100 µl of extracted solution was transferred to a 96-well microtiter plate and the optical density was measured at 560 nm in a microplate reader (SH-9000, Corona Electric).

### Immunofluorescence staining

ICC cells transfected with siRNA in a chamber slide (SCS-N02, Matsunami Glass Ind. Ltd., Osaka, Japan) were fixed with a 10% neutral buffered formalin for 10 min, and treated with 0.05% Triton X-100™ (T8787, Merk Sigma-Aldrich) in phosphate-buffered saline for 5 min. Then, cells were treated with 10% normal goat (ab7481, Abcam) and donkey serum (ab7475, Abcam) in phosphate-buffered saline for 30 min. Cells were then incubated with the following primary antibodies; SMYD2 (sc-393827, Santa Cruz Biotechnology, 1:50) and CCR7 (25898-1-AP, Proteintech, 1:200) for 1 h. After the primary antibody reaction, cells were incubated with secondary antibodies; donkey anti-mouse IgG H&L Alexa Fluor^®^ 594 (ab150108, Abcam, 1:1000) and goat anti-rabbit IgG H&L Alexa Fluor^®^ 488 (ab150077, Abcam, 1:1000) for 1 h. Cells were then treated with 4’6-diamidino-2-phenylindole solution (#340–07971, Dojindo, 1:1000) for 1 min and observed using a fluorescence microscope BZ-X800 (Keyence, Osaka, Japan). All procedures were performed at room temperature.

### Statistics

Statistical analysis was performed using JMP ver.18 (SAS Institute, Cary, NC). Fisher’s exact test and the Mann-Whitney *U* test were used to analyze clinicopathological data. The Kaplan-Meier method was used to analyze OS and disease free survival (DFS), and the log-rank test was used to determine the statistical significance. Cox proportional hazards model was used to evaluate the risk factors for OS and DFS. Spearman’s correlation analysis was used to investigate the relationship between SMYD2 and CCR7 expression levels on the metastatic tumor cells in LNs. All experiments were performed in triplicate, and Student’s *t*-test was used for statistical analysis. Statistical significance was defined as *p* < 0.05.

## Results

### Expression levels of SMYD2 in the primary lesion were not associated with ICC prognosis

**Fig. 1:**
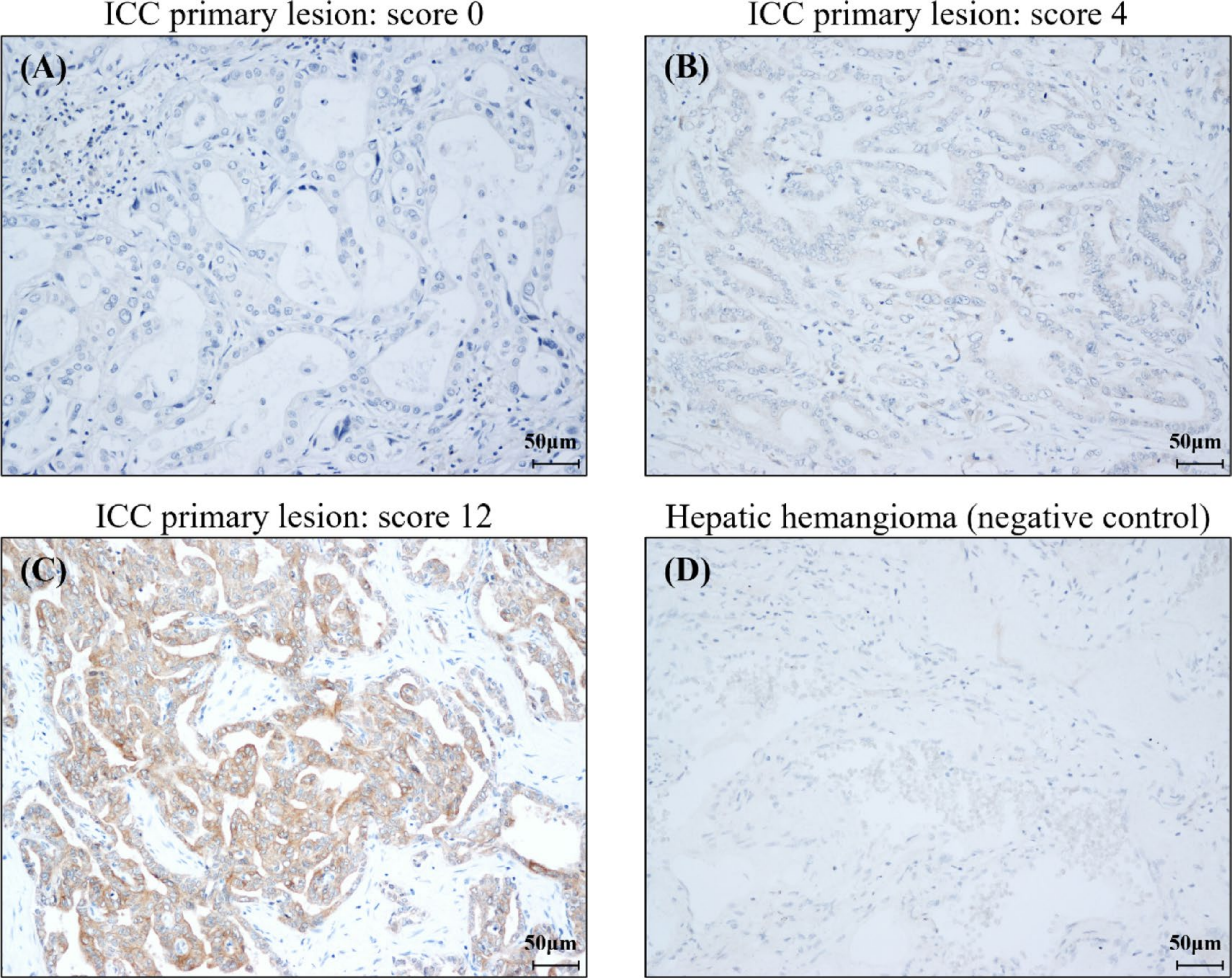
Representative micrographs of immunohistochemistry of SMYD2 in primary ICC scored by IRS. (**A**) primary lesion: score 0 (**B**) primary lesion: score 4 (**C**) primary lesion: score 12 (**D**) hepatic hemangioma as a negative control. (magnification-x200, scale bar means 50 μm).

**Fig. 2:**
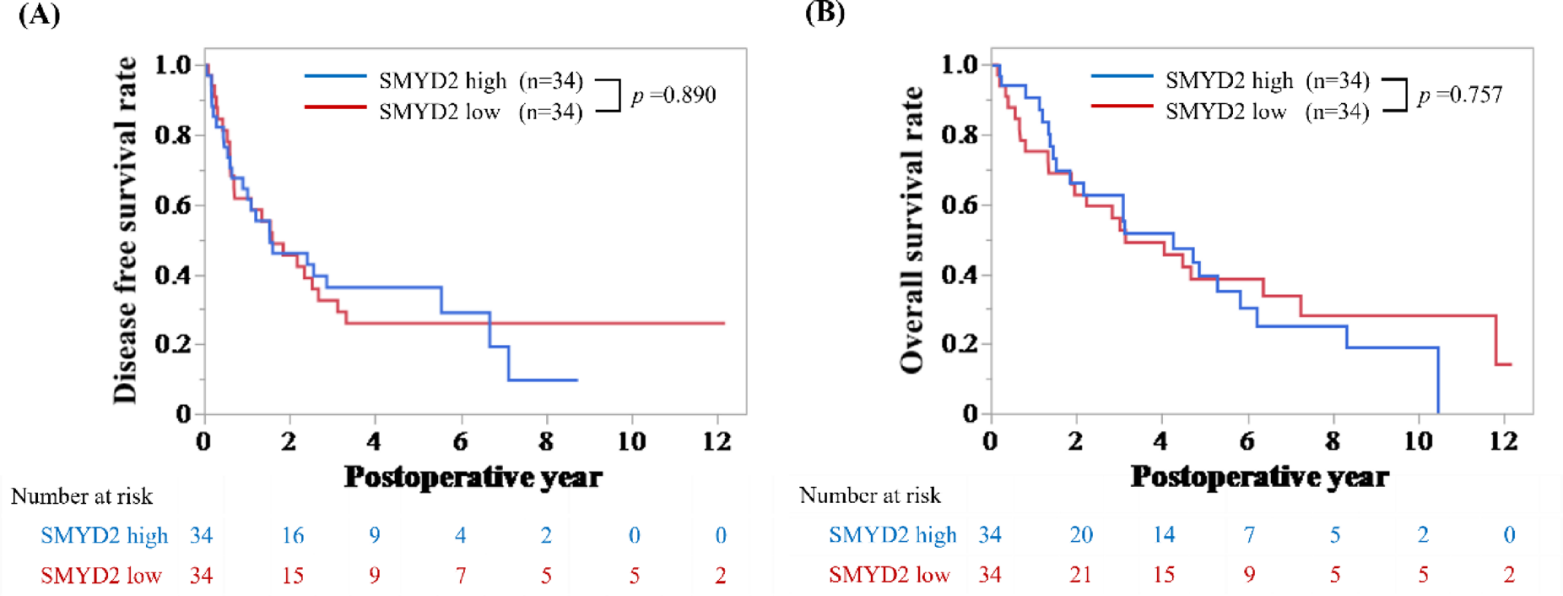
Kaplan-Meier curves for DFS and OS showing high vs. low SMYD2 expression in primary ICC. (**A**) DFS in cases with high vs. low SMYD2 expression. (**B**) OS in cases with high vs. low SMYD2 expression. The log-rank test was used for statistical analysis. *p* < 0.05 is considered to be statistically significant.

All cases in the primary ICC lesion were categorized into groups according to the cut-off value set at the median IRS = 3.83: SMDY2-high (IRS ≥ 3.83; *n* = 34) or SMYD2-low group (IRS < 3.83, *n* = 34). Representative slides of SMYD2 expression in the primary liver tumor based on each score are shown in Fig. 1. Clinicopathological characteristics of SMYD2 high and low groups are shown in Tables 1 and 2. There was no statistically significant difference between these two groups among all variables, including LNM. SMYD2 expression in the primary lesion was not significantly associated with DFS (*p* = 0.890) or OS (*p* = 0.757), as shown on Kaplan-Meier curves (Fig. 2A and B). Univariate analysis showed that SMYD2 expression was not associated with DFS (*p* = 0.89), whereas age < 65 years old, carbohydrate antigen 19 − 9 (CA19-9) ≥ 38 u/ml, tumor size ≥ 5 cm, lower differentiation, portal venous invasion and pathological LNM were significantly associated with poor DFS, and multivariate analysis revealed that age < 65 years old, portal venous invasion and pathological LNM were significantly associated with poor DFS (Table 3). Furthermore, univariate analysis showed that SMYD2 expression was not associated with OS (*p* = 0.76), whereas CA19-9 ≥ 38 u/ml, portal venous invasion and pathological LNM were significantly associated with poor OS, and multivariate analysis revealed that portal venous invasion and pathological LNM were significantly associated with poor OS (Table 4).

**Table 1:** Patients’ characteristics depend on SMYD2 expression in the primary lesion with clinical factors.

Variables		All (*n* = 68)	SMYD2 high expression (*n* = 34)	SMYD2 low expression (*n* = 34)	*p*- value
Sex					0.21
	Male	42	24	18	
	Female	26	10	16	
Age (years)		68 (40–85)	66.5 (40–81)	69 (54–85)	0.11
Hepatitis status					0.24
	HBV Ag/HCV Ab positive	15	10	5	
	No infection	53	24	29	
Preoperative laboratory data					
	Albumin (g/dl)	4.2 (2.7–5)	4.25 (3–5)	4.2 (2.7–4.8)	0.52
	Total bilirubin (mg/dl)	0.8 (0.2–11.3)	0.75 (0.2–1.4)	0.8 (0.3–11.3)	0.12
	Prothrombin time (%)	94.1 (60.7–118)	94.1 (63.3–111.9)	94.1 (60.7–118)	0.64
	ICG R15 (%)	0.16 (0.043–0.29)	0.16 (0.12–0.24)	0.16 (0.043–0.29)	0.40
	AST (IU/ml)	29 (12–287)	29 (17–128)	29.5 (12–287)	0.78
	ALT (IU/ml)	27 (8–634)	25.5 (8–139)	30.5 (10–634)	0.31
	Platelet count (x10^4^/µl)	20.95 (8.1–80.3)	22.8 (9.5–80.3)	20.8 (8.1–36.3)	0.25
	CEA (ng/ml)	2.7 (0.1–396)	2.1 (0.1–27.2)	3.8 (0.2–396)	0.21
	CA19-9 (U/ml)	56.6 (1.2–252025.4)	45.2 (1.2–117827.3)	88 (2–252025.4)	0.45
Child-Pugh grade					0.36
	A	66	34	32	
	B	1	0	1	
	C	1	0	1	

*SMYD2* SET and MYND domain-containing 2, *HBV* hepatitis B virus, *Ag* antigen, *HCV* hepatitis C virus, *Ab* antibody, *ICG R15* indocyanine green retention test at 15 min, *AST* aspartate transaminase, *ALT* alanine transaminase, *CEA* carcinoembryonic antigen, *CA19-9* carbohydrate antigen 19−9.

**Table 2:** Patients’ characteristics depend on SMYD2 expression in primary lesion with pathological characteristics.

Variables		All (*n* = 68)	SMYD2 high expression (*n* = 34)	SMYD2 low expression (*n* = 34)	*p*- value
Tumor maximum size (cm)		3.7 (0.8–15)	4 (2.3–9)	3 (0.8–15)	0.53
Tumor number					0.19
	Single	57	26	31	
	Multiple	11	8	3	
Differentiation					0.74
	Well	11	6	5	
	Moderate/poor	54	25	29	
	Undefined	3	3	0	
Portal venous invasion					0.31
	Present	25	10	15	
	Absent	43	24	19	
Hepatic venous invasion					1.00
	Present	13	7	6	
	Absent	55	27	28	
Arterial invasion					1.00
	Present	3	1	2	
	Absent	65	33	32	
Biliary invasion					0.46
	Present	28	12	16	
	Absent	40	22	18	
LNM					1.00
	Present	15	7	8	
	Absent	53	27	26	

*SMYD2* SET and MYND domain-containing 2, *LNM* lymph node metastasis. Statistically significant values are in bold.

**Table 3:** Univariate and multivariate analysis for disease-free survival of ICC.

Variables	Univariate analysis			Multivariate analysis		
	HR	(95% CI)	*p*-value	HR	(95% CI)	*p*-value
Sex (male vs. female)	1.15	0.63–2.09	0.65			
Age (≥ 65 vs. <65)	0.55	0.31–0.995	**0.048**	0.51	0.26–0.97	**0.042**
Viral infection (yes vs. no)	0.92	0.46–1.86	0.83			
CEA (≥ 5 vs. <5 ng/ml)	1.25	0.68–2.33	0.47			
CA19-9 (≥ 38 vs. <38 u/ml)	2.38	1.28–4.44	**0.006**	1.83	0.91–3.68	0.090
Tumor size (≥ 5 vs. <5 cm)	2.23	1.23–4.06	**0.008**	1.57	0.81–3.06	0.18
Tumor number (multiple vs. single)	1.22	0.59–2.53	0.60			
Differentiation (mod/por vs. wel)	3.67	1.31–10.34	**0.013**	1.36	0.43–4.27	0.60
Portal venous invasion (yes vs. no)	3.62	1.95–6.73	**< 0.0001**	5.39	2.69–10.80	**< 0.0001**
Hepatic venous invasion (yes vs. no)	1.24	0.61–2.49	0.55			
Arterial invasion (yes vs. no)	0.96	0.23–3.97	0.96			
Pathological LNM (yes vs. no)	3.15	1.61–6.18	**0.0008**	3.79	1.72–8.32	**0.0009**
Adjuvant chemotherapy (yes vs. no)	1.39	0.78–2.48	0.26			
SMYD2 (high vs. low)	1.04	0.59–1.85	0.89			

*ICC* intrahepatic cholangiocellular carcinoma, *HR* hazard ratio, *CI* confidence interval, *CEA* carcinoembryonic antigen, *CA19-9* carbohydrate antigen 19-9, *LNM* lymph node metastasis, *SMYD2* SET and MYND domain-containing 2. Statistically significant values are in bold.

**Table 4:** Univariate and multivariate analysis for overall survival of ICC.

Variables	Univariate analysis			Multivariate analysis		
	HR	(95% CI)	*p*-value	HR	(95% CI)	*p*-value
Sex (male vs. female)	1.42	0.76–2.67	0.27			
Age (≥ 65 vs. <65)	0.73	0.39–1.36	0.32			
Viral infection (yes vs. no)	0.80	0.38–1.68	0.56			
CEA (≥ 5 vs. <5 ng/ml)	1.48	0.78–2.81	0.23			
CA19-9 (≥ 38 vs. <38 u/ml)	2.02	1.08–3.78	**0.03**	1.75	0.91–3.36	0.09
Tumor size (≥ 5 vs. <5 cm)	1.80	0.97–3.35	0.06			
Tumor number (multiple vs. single)	1.41	0.69–2.86	0.35			
Differentiation (mod/por vs. wel)	1.81	0.80–4.11	0.15			
Portal venous invasion (yes vs. no)	3.25	1.73–6.11	**0.0002**	3.44	1.79–6.62	**0.0002**
Hepatic venous invasion (yes vs. no)	1.84	0.89–3.79	0.10			
Arterial invasion (yes vs. no)	1.35	0.32–5.64	0.28			
Pathological LNM (yes vs. no)	3.51	1.74–7.10	**< 0.001**	4.47	2.13–9.41	**< 0.0001**
Adjuvant chemotherapy (yes vs. no)	1.03	0.56–1.88	0.92			
SMYD2 (high vs. low)	1.10	0.60–2.00	0.76			

*ICC* intrahepatic cholangiocellular carcinoma, *HR* hazard ratio, *CI* confidence interval, *CEA* carcinoembryonic antigen, *CA19-9* carbohydrate antigen 19-9, *LNM* lymph node metastasis, *SMYD2* SET and MYND domain-containing 2. Statistically significant values are in bold.

### LNM derived from ICC was associated with poor prognosis and SMYD2 expression

**Fig. 3:**
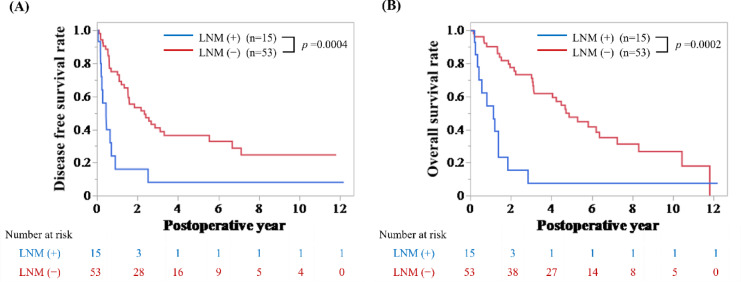
Kaplan-Meier curves for DFS and OS showing positive vs. negative LNM in ICC. (**A**) DFS in cases with positive vs. negative LNM. (**B**) OS in cases with positive vs. negative LNM. The log-rank test was used for statistical analysis. *p* < 0.05 is considered statistically significant.

**Fig. 4:**
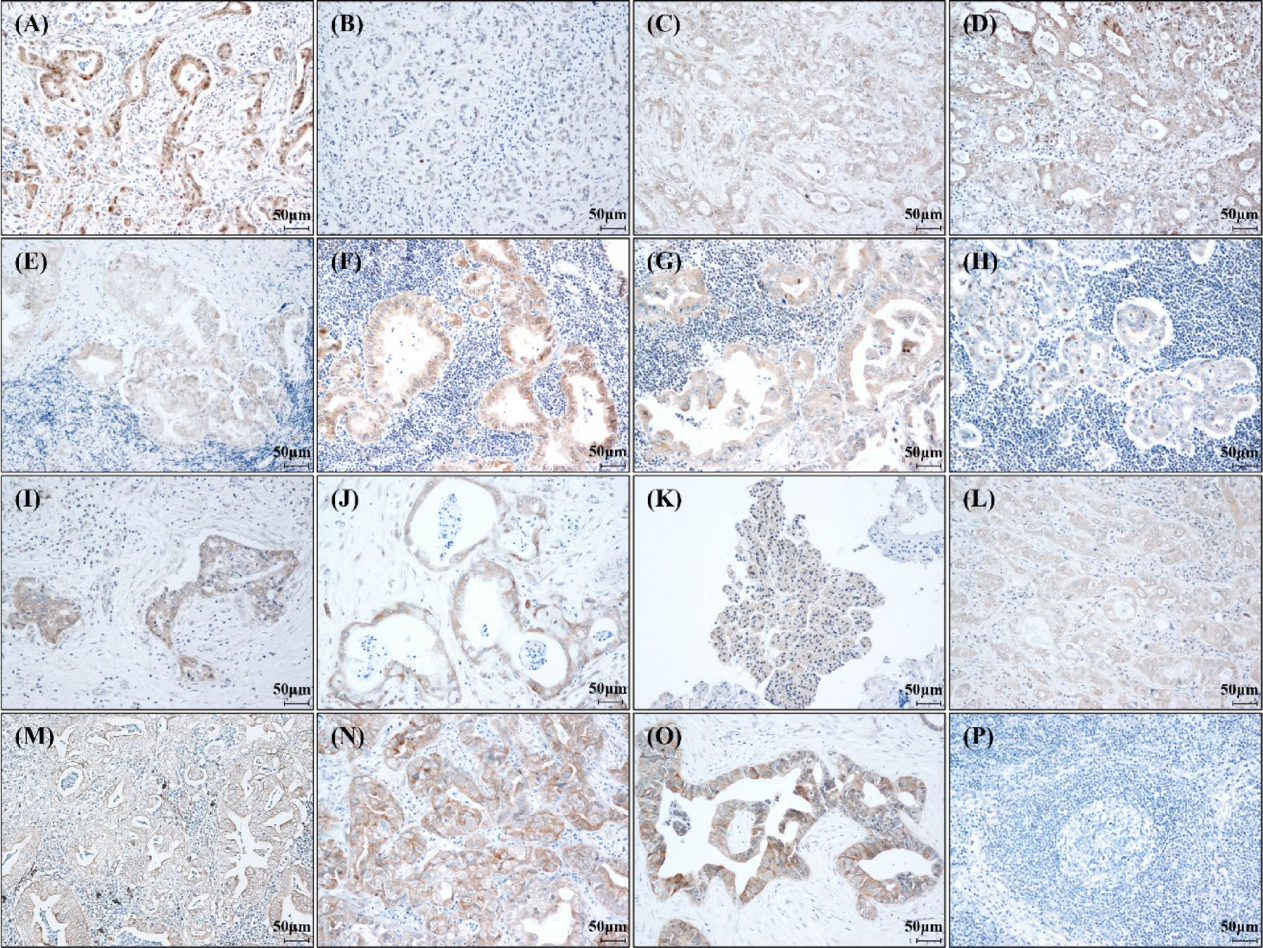
Immunohistochemistry of SMYD2 in metastatic LNs derived from ICC of all 15 cases. (**A**)–(**O**) SMYD2 was expressed in all LNM cases derived from ICC. The alphabet attached to each case was the same as in Fig. 7. (**P**) A LN without metastasis from ICC as a negative control. (magnification-x200, scale bar means 50 μm).

Expression level of SMYD2 in the primary lesion did not affect either OS or DFS, but LNM was strongly associated with both poor OS and poor DFS. In our models, 15 cases were diagnosed as pathologically positive LNM at the point of surgery, and they had significantly poorer OS and DFS than the cases without LNM, as demonstrated by Kaplan-Meier curves (Fig. 3A and B). Therefore, we next investigated whether SMYD2 was involved in LNM. Immunohistochemistry of metastatic LNs revealed that metastatic cells expressed SMYD2 in all cases, although with varying expression levels (Fig. 4A-O). On the other hand, SMYD2 expression was not observed in non-metastatic LNs (Fig. 4P).

### SMYD2 regulated ICC cell proliferation

**Fig. 5:**
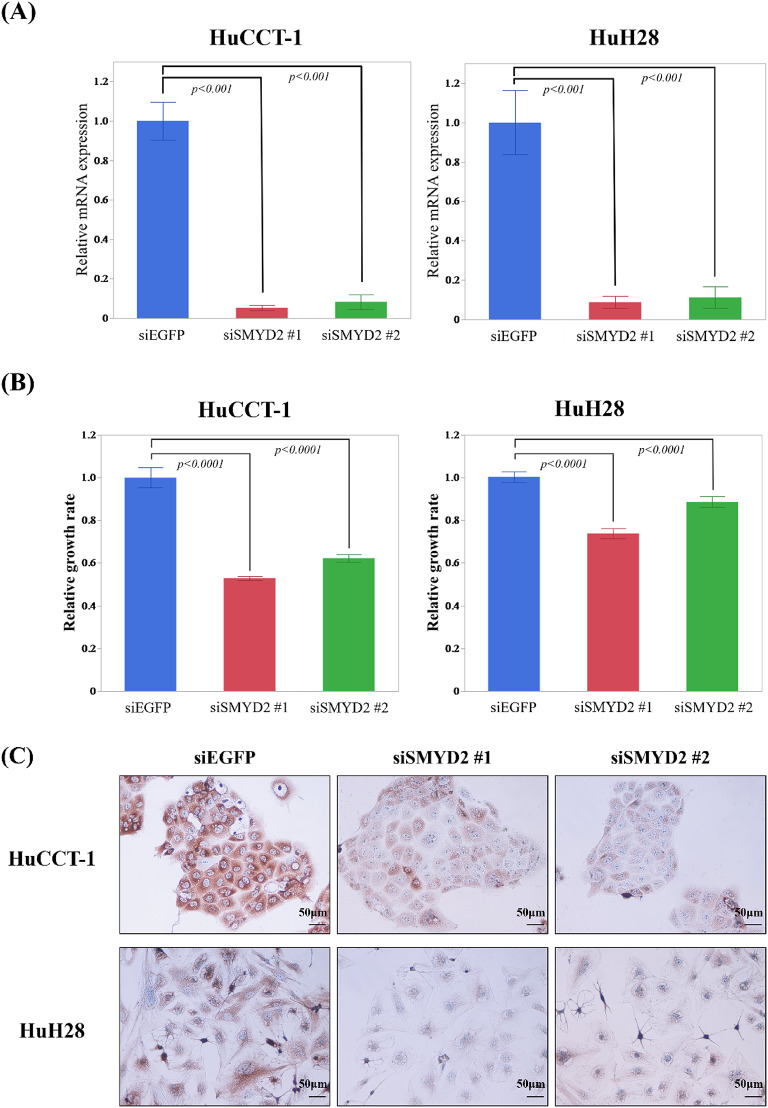

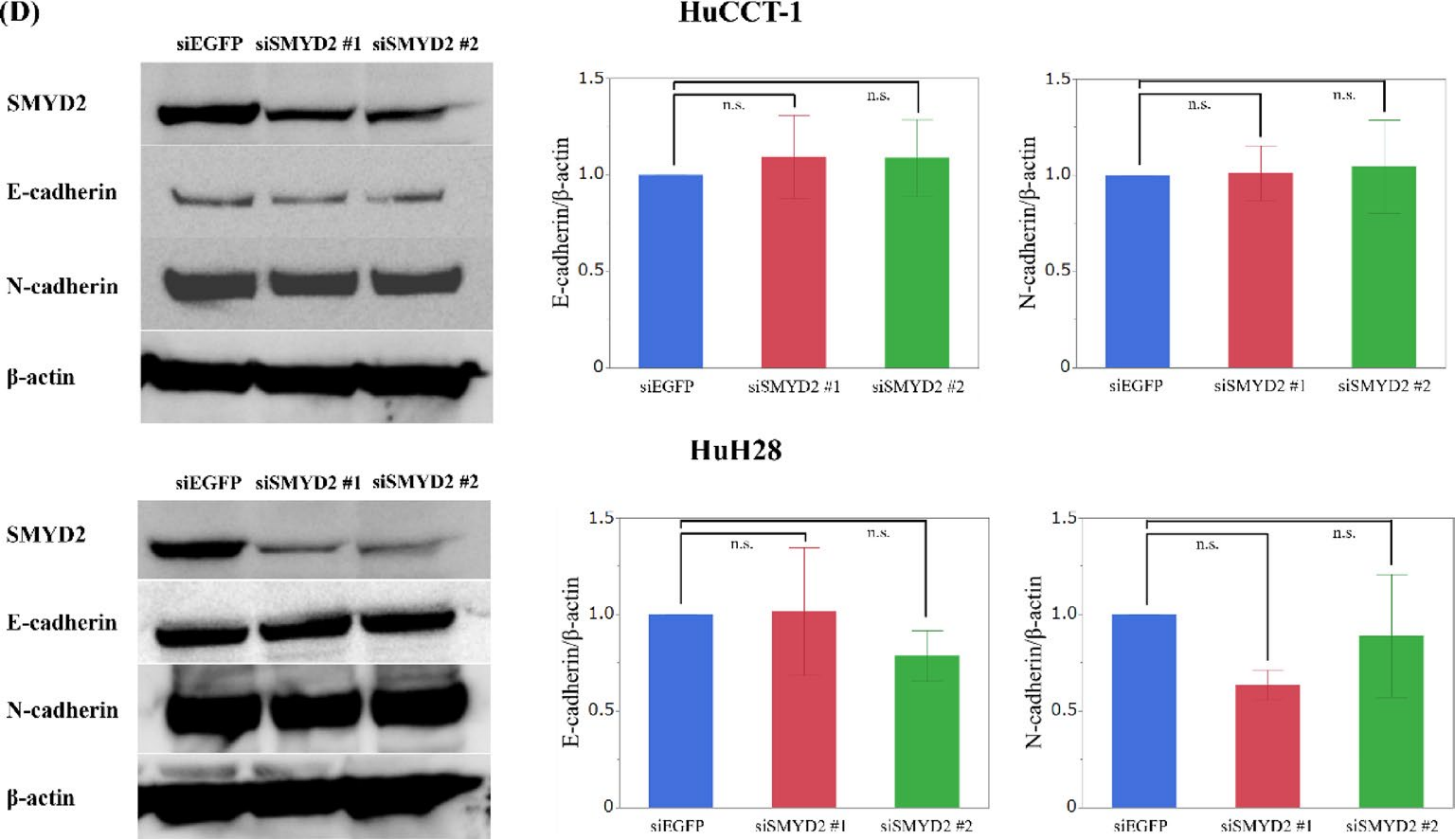
EMT related analysis using siRNA specific for SMYD2 in HuCCT-1 and HuH28. (**A**) Relative messenger RNA expression level by qRT-PCR in siEGFP and siSMYD2. siSMYD2 significantly suppressed SMYD2 expression at the mRNA level compared to siEGFP. (**B**) Cell proliferation analysis in siEGFP and siSMYD2. Cell proliferation was suppressed by siSMYD2 relative to siEGFP in both HuCCT-1 and HuH28. (**C**) Immunocytochemistry of SMYD2 on HuCCT-1 and HuH28 treated with siEGFP or siSMYD2. Although siSMYD2 suppressed SMYD2 expression at the protein level, no morphological changes were observed in both cell lines compared to siEGFP. (magnification-x200, scale bar means 50 μm). (**D**) Cadherin switching was evaluated by western blotting using anti E- and N-cadherin antibody. siSMYD2 suppressed SMYD2 expression, whereas cadherin switching was not induced in both cell lines. Original blots are presented in Supplementary Fig. S1 and S2, and the cropped blots enclosed by squares in those files are shown. *p* < 0.05 is considered statistically significant.

Expression pattern of SMYD2 in LNM suggested that SMYD2 was one of the regulators in the mechanism of LNM. We therefore sought to elucidate the role of SMYD2 for LNM in a stepwise manner by loss-of-function analysis of SMYD2. SMYD2-specific siRNAs transfected to HuCCT-1 and HuH28 suppressed SMYD2 expression at both mRNA and protein levels, compared with the negative control (siEGFP) (Fig. 5A and D). Cell proliferation assay revealed that knockdown of SMYD2 expression significantly inhibited cell proliferation in both cell lines (Fig. 5B), suggesting that SMYD2 may contributes to the establishment of metastatic lesions.

### Morphological changes and cadherin switching were not induced by SMYD2 knockdown

To assess whether SMYD2 was associated with EMT as the main mechanism for the metastasis^32^, we evaluated the morphological changes and cadherin switching, a process of downregulation of E-cadherin and upregulation of N-cadherin^34–36^. Despite the inhibition of SMYD2 expression, cell morphology and motility of siSMYD2 transfected cells were similar to controls in both cell lines (Fig. 5C). Furthermore, although the representative image might visually suggest a slight decrease E-cadherin expression in HuCCT-1, no statistically significant difference was observed in E-cadherin or N-cadherin expression in either siEGFP or siSMYD2 (Fig. 5D, Supplementary Fig. S1 and S2). Taken together, it was suggested that SMYD2 might not regulate LNM through EMT pathway, which was well-known as the metastatic mechanism.

### SMYD2 regulated chemotaxis of cells derived from metastatic loci but not from primary ICC

**Fig. 6:**
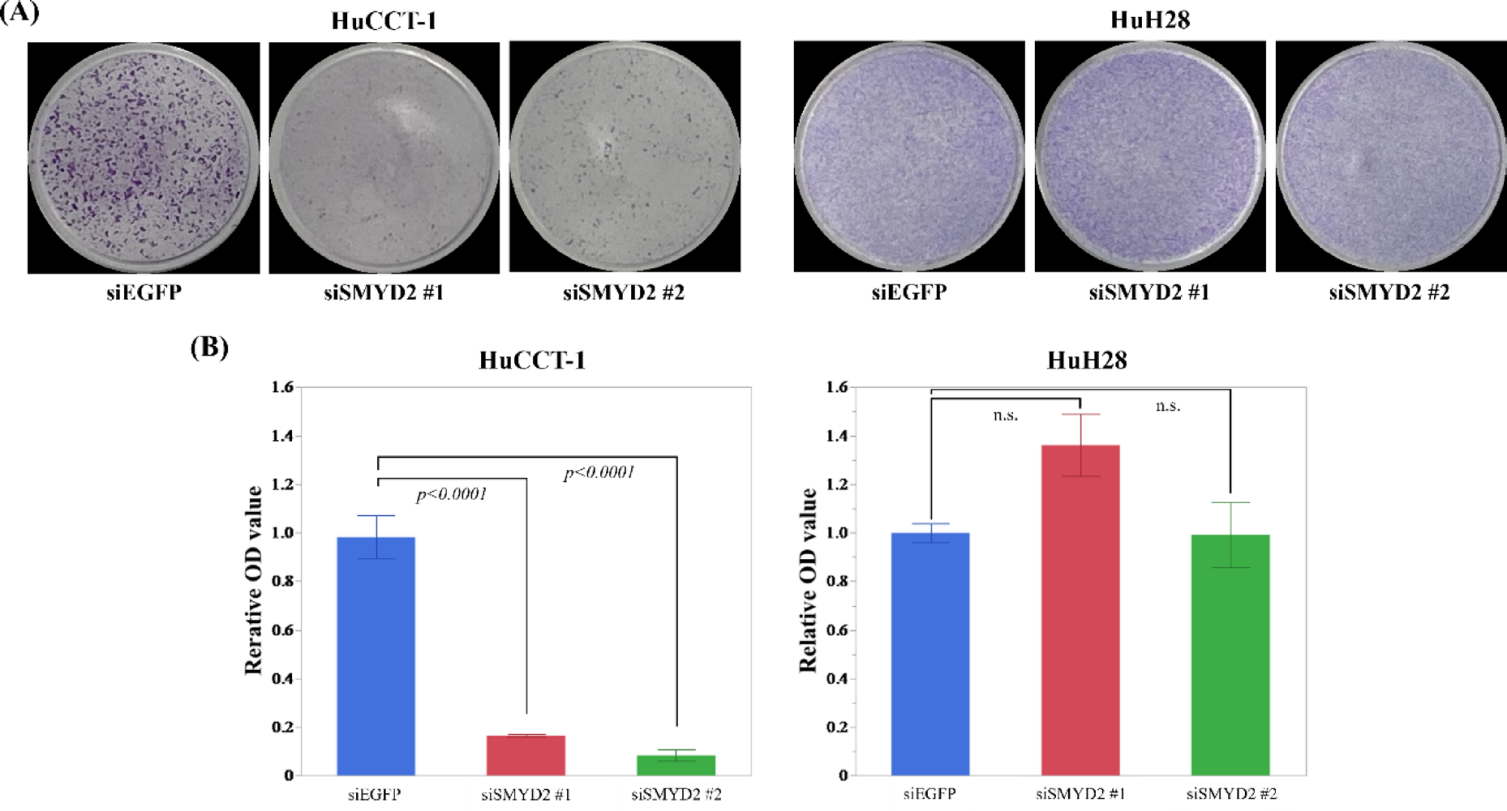
Chemotaxis assay using SMYD2-specific siRNAs in HuCCT-1 and HuH28. (**A**) Macroscopic observation of each transwell chamber. (**B**) Absorption measured at 560 nm. Cellular chemotaxis was significantly suppressed by siSMYD2 transfection in HuCCT-1, but not in HuH28. *p* < 0.05 is considered statistically significant.

The metastatic pathway was considered unlikely to involve EMT, then we therefore performed chemotaxis assay to evaluate the influence of SMYD2 on the chemotactic abilities of ICC. In HuCCT-1, which was established from malignant ascites cells, there was considerable suppression of chemotactic migration of siSMYD2 transfected cells compared with that of siEGFP. Conversely in HuH28, which was derived from the primary ICC lesion, there was no difference in chemotactic migration between siSMYD2 and siEGFP transfected cells (Fig. 6).

### SMYD2 and CCR7 expression levels in metastatic LNs were positively correlated

**Fig. 7:**
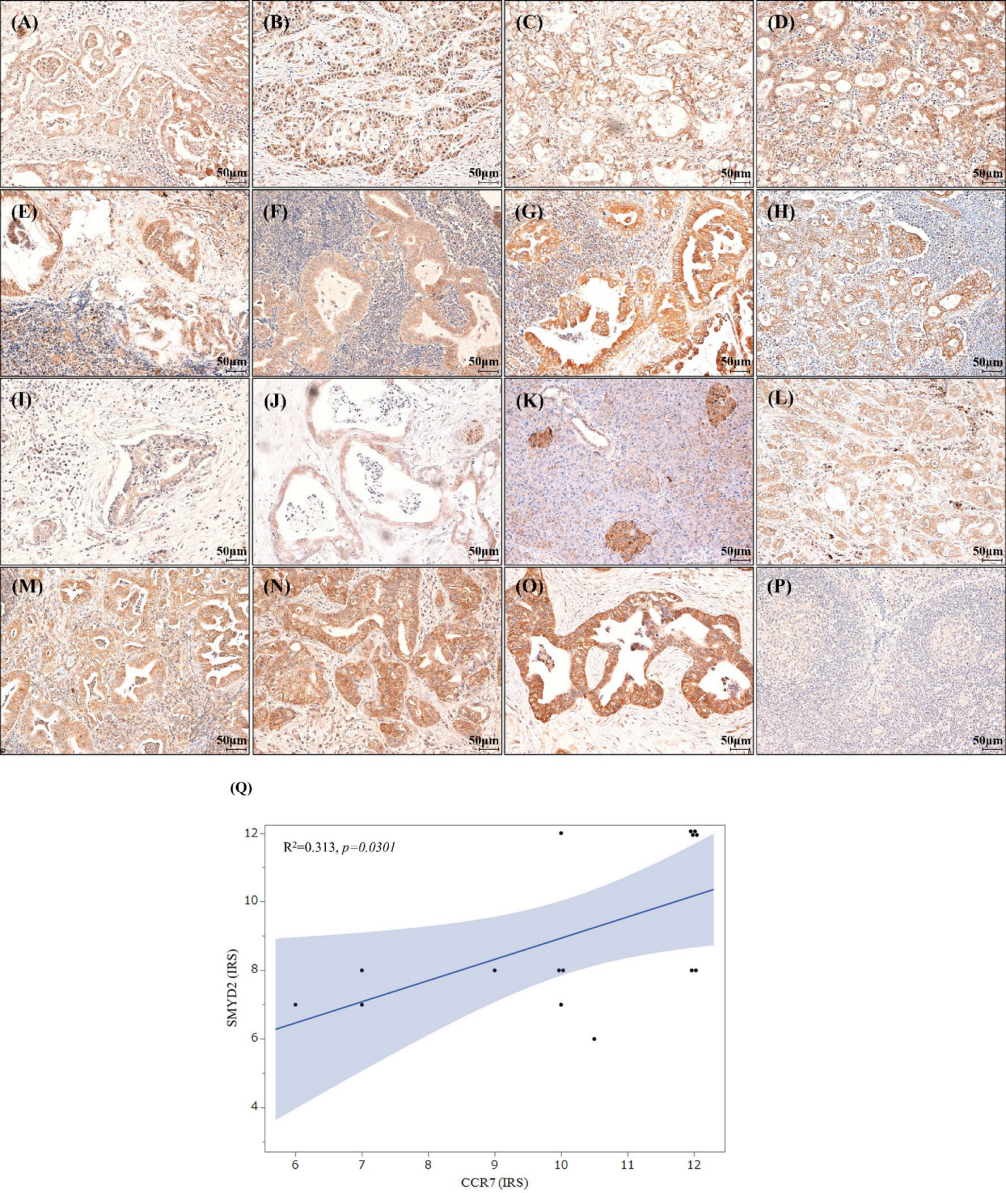
Immunohistochemistry of CCR7 in metastatic LNs derived from ICC of all 15 cases. (**A**)–(**O**) CCR7 was expressed in all LNM cases derived from ICC as well as SMYD2. The alphabet attached to each case was the same as in Fig. 4. (**P**) A LN without metastasis from ICC as a negative control. (magnification-x200, scale bar means 50 μm). (**Q**) Spearman’s correlation analysis between SMYD2 and CCR7 expression levels in the metastatic tumor cells of LNs. *p* < 0.05 is considered statistically significant.

Next, we conducted the immunohistochemistry of CCR7, one of the classic molecules mediating chemotaxis, in clinical metastatic LNs. Chemotaxis is induced through the chemokine-chemokine receptor axis. CCR7 plays an important role in cellular migration to LNs, and overexpression of CCR7 was reportedly associated with LNM in various malignancies^37–41^. CCR7 expression in metastatic LN cells was also confirmed in all cases (Fig. 7A-O), as was SMYD2. Based on the results that both SMYD2 and CCR7 were expressed at the metastatic lesion in all cases, we analyzed the correlation of their expressions using IRS. Spearman’s rank correlation analysis revealed a significant positive correlation between SMYD2 and CCR7 (Fig. 7Q. *p* = 0.031).

### SMYD2 regulated CCR7 expression of HuCCT-1

**Fig. 8:**
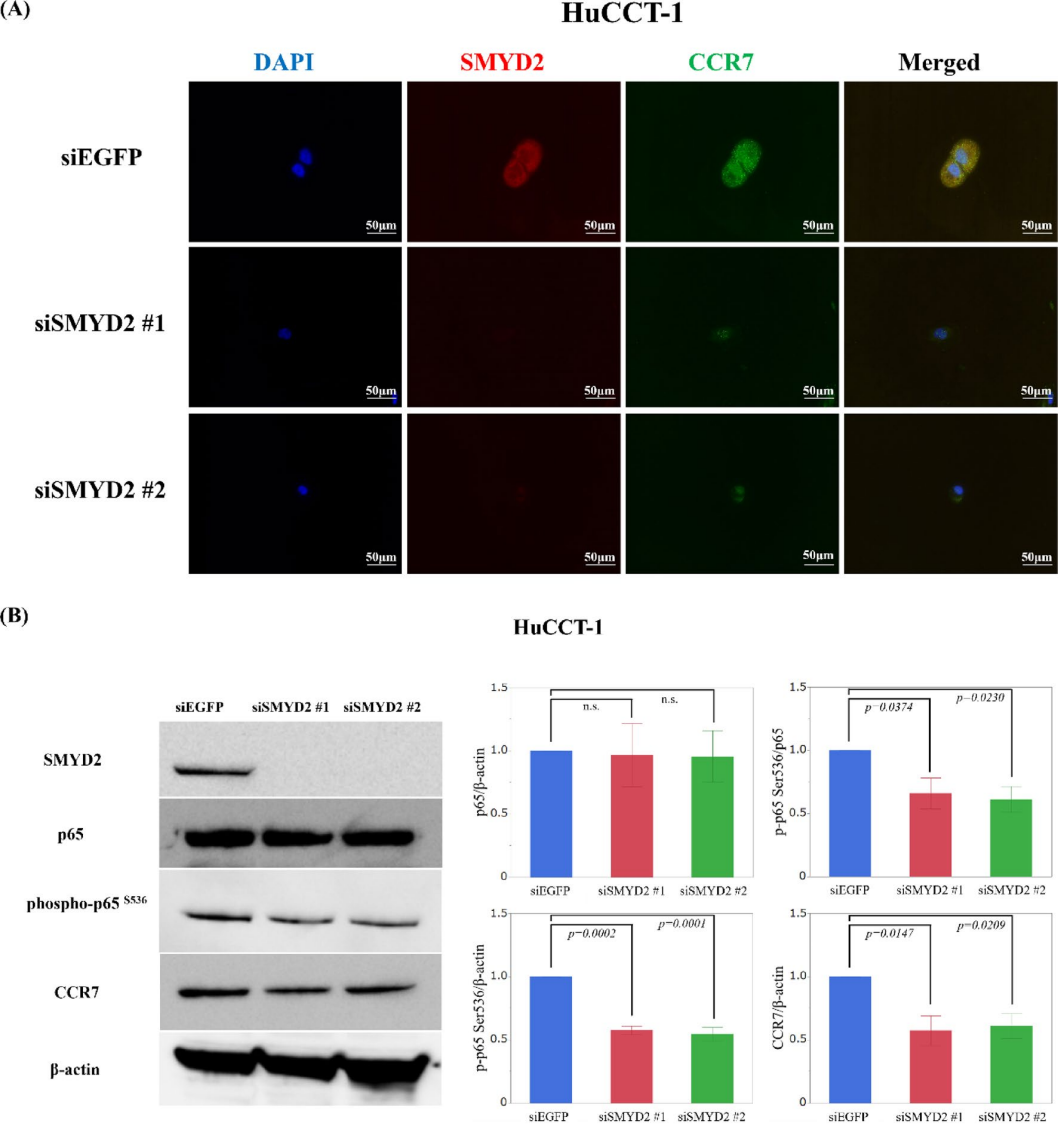
Immunofluorescence staining of CCR7 and western blotting of CCR7, p65 and phosphor-p65^S536^ using siSMYD2 or siEGFP transfected HuCCT-1. (**A**) siSMYD2 suppressed both SMYD2 and CCR7 expressions compared to siEGFP. (magnification-x400, scale bar means 50 μm). (**B**) siSMYD2 suppressed CCR7 expression with attenuated phosphorylation of p65^S536^ without altering p65 expression relative to siEGFP. *p* < 0.05 was considered statistically significant. Original blots are presented in Supplementary Fig S3, and the cropped blots enclosed by squares in this file are shown.

To investigate the biological relationship between SMYD2 and CCR7, we evaluated the CCR7 expression in siRNA-transfected HuCCT-1. Immunofluorescence staining and western blotting revealed that the expression of CCR7 was downregulated in the siSMYD2 transfected HuCCT-1 compared with that of siEGFP (Fig. 8 and Supplementary Fig. S3).

### SMYD2 regulated the phosphorylation of p65^S536^

CCR7 expression was reportedly enhanced by NF-κB^42–44^, so we focused on the NF-κB pathway to investigate the mechanism by which SMYD2 regulates CCR7 in ICC. Western blotting of CCR7, p65 and phospho-p65^S536^ using HuCCT-1 revealed CCR7 attenuation with reduced phosphorylation of p65^S536^ in the siSMYD2 transfected HuCCT-1 compared with that of siEGFP, even though the expression of p65 did not differ in either siEGFP or siSMYD2 (Fig. 8B and Supplementary Fig. S3).

## Discussion

We demonstrated that SMYD2 was overexpressed in metastatic LN, but not in primary ICC lesions. Overexpression of SMYD2 in primary lesions of various malignant tumors has been associated with tumor progression and poor prognosis^28–31^. We therefore hypothesized that overexpression of SMYD2 in ICC primary lesion may also contributes to tumor progression and poor prognosis. To explore this hypothesis, we evaluated the relationship between the SMYD2 expression in ICC primary lesion and clinicopathological parameters including DFS and OS, but we found no significant correlation. Next, LNM from ICC is clinically associated with poor prognosis^1–7^, we therefore investigated the SMYD2 expression levels in metastatic and non-metastatic LNs. As a result, SMYD2 expression was observed in metastatic LN cells of all cases, but not in non-metastatic. SMYD2 was thus indicated to be potentially involved in LNM derived from ICC, so we subsequently sought to elucidate the role that SMYD2 plays in LNM from ICC.

The establishment of LNM consists of the following processes: lymphangiogenesis, formation of pre-metastatic niche, tumor cell migration into lymphatic duct and LNs, and immune evasion in metastatic LNs and metabolic adaptation of tumor cells in LNs^45,46^. Among these processes, we particularly focused on tumor cell migration because this process is the most fundamental and essential steps for metastatic cells. Without this process, subsequent events in LNs including evasion from immune cells and metabolic adaptation, could not occur. To evaluate the role of SMYD2 in this process, based on the previous report in colorectal cancer^32^, we first investigated whether SMYD2 regulated EMT. We then assessed the morphological changes and cadherin switching following SMYD2 knockdown. Cadherin switching, downregulation of E-cadherin and upregulation of N-cadherin, and morphological changes, which transform compact and adherent cells into elongated and motile phenotypes, are often observed in more aggressive cells and are associated with dissemination and metastasis. Our research nonetheless showed that SMYD2 was unlikely to be involved in LNM of ICC causing EMT, because SMYD2 knockdown did not cause the morphological changes and cadherin switching. Next, we therefore investigated the effect of SMYD2 on the chemotaxis that plays an important role through cellular homing to LNs and invasion into LNs from lymphatic vessels through various chemokine-chemokine receptor axes.

At first, we revealed that SMYD2 regulated the chemotaxis of ICC cells derived from metastatic loci but not from the primary tumor in chemotaxis assay. These results suggested the potential involvement of SMYD2 in metastasis establishment through chemotaxis, and we focused on the C-C motif chemokine ligand (CCL) 19/CCL21-CCR7 axis, which plays an important role in cellular migration to LNs. CCL19 and CCL21 were constitutively expressed in LNs, and induced homing and invasion of cells expressing their receptor, CCR7^47^. CCR7 overexpression was reportedly associated with LNM and poor prognosis in esophageal squamous cell carcinoma^37,38,41^, gastric cancer^39^ and breast cancer^40^. We immunohistochemically revealed that CCR7 was expressed in metastatic LN cells of all cases, as well as SMYD2, and there was a significant positive correlation between SMYD2 and CCR7 expression. Therefore, we subsequently investigated the biological relationship between these two molecules.

**Fig. 9:**
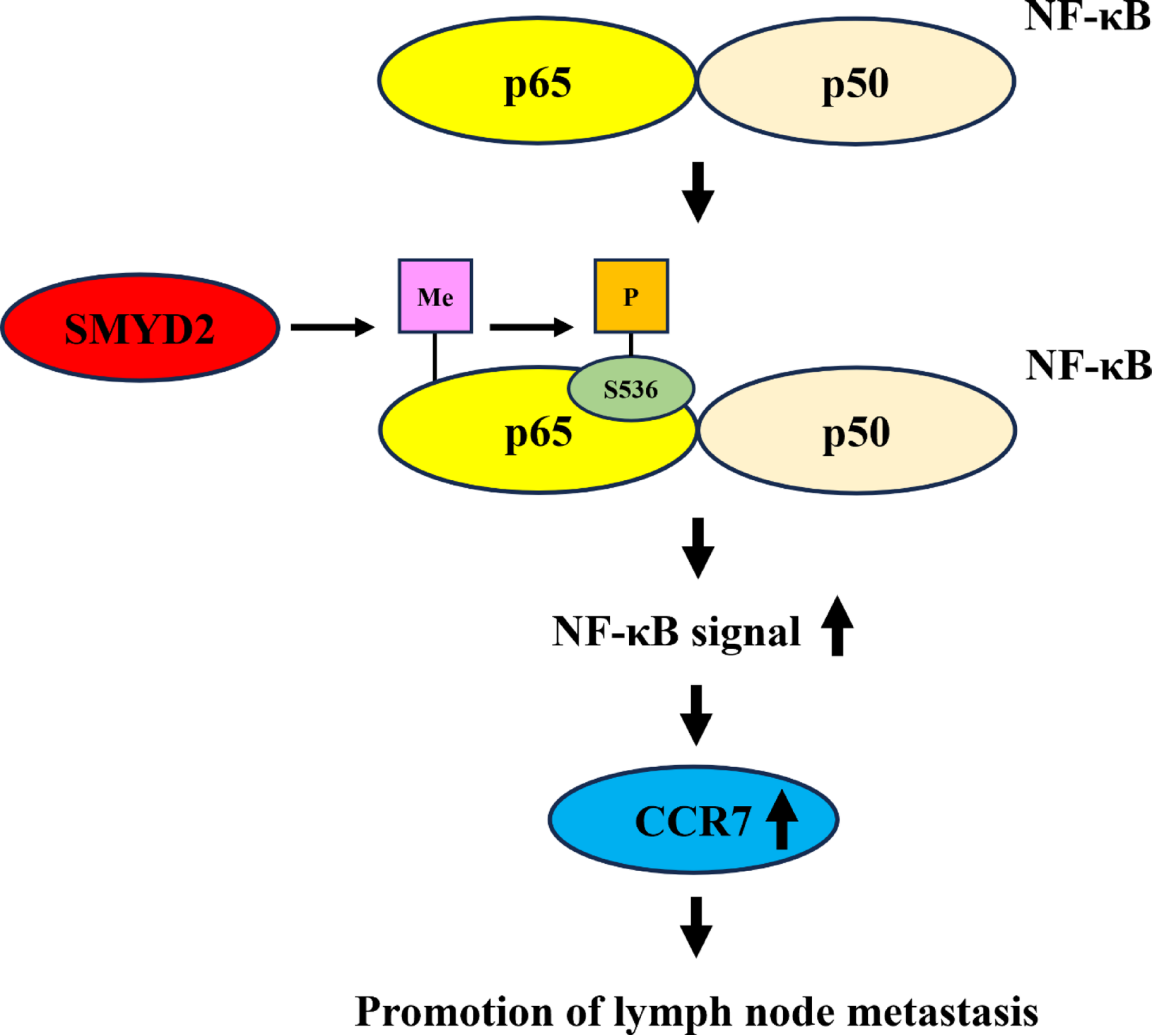
Schematic model for the regulation of CCR7 by SMYD2, SMYD2-phospho-p65^S536^-CCR7 pathway. Methylation of p65 of NF-κB by SMYD2 induces phosphorylation of p65^S536^ without reducing p65 expression, subsequently activating NF-κB signal. This enhances CCR7 expression and finally promotes LNM.

In the subsequent immunofluorescence staining and western blotting, CCR7 expression of HuCCT-1 was shown to be suppressed by SMYD2 knockdown, which might indicate the involvement of CCR7 expression in the cellular chemotaxis. CCR7 has been reported to be upregulated by NF-κB, so we investigated the mechanism of CCR7 regulation by SMYD2 focusing on NF-κB pathway^42–44^. Furthermore, SMYD2 has reportedly methylated p65 of NF-κB and increased phosphorylation of p65^S536^, which resulted in activation of NF-κB in triple negative breast cancer^30^. We therefore examined the relationship between CCR7 and p65 expression levels and phosphorylation level of p65^S536^ following SMYD2 suppression. SMYD2 knockdown reduced CCR7 expression and phosphorylation level of p65^S536^ without reducing p65 expression. SMYD2 was indicated by these results to be involved in the cellular chemotaxis through the SMYD2-phospho-p65^S536^-CCR7 pathway (Fig. 9). The individual mechanisms connecting SMYD2 to NF-κB, NF-κB to CCR7, and CCR7 to chemotaxis have been previously reported. However, our study provided both clinical and biological evidence for the integration of this entire signaling pathway in the specific context of LNM derived from ICC. Notably, our findings suggested that SMYD2 predominantly contributed to LNM by facilitating CCR7-dependent chemotaxis rather than inducing EMT, thereby offering a novel mechanistic insight into ICC progression.

This study has several limitations. We could not demonstrate the clinical importance of SMYD2 in primary ICC, and there might be reasons for the small numbers of ICC cases. Second, there is still insufficient demonstration of how the phosphorylation of p65^S536^, which is induced by SMYD2-mediated methylation of p65 within the SMYD2-phospho-p65^S536^-CCR7 pathway, regulates CCR7 expression. Finally, although our *in vitro* data demonstrated that SMYD2 knockdown reduced the chemotactic ability of metastatic ICC cells, determining the direct causal necessity of CCR7 requires further rescue experiments. Furthermore, accurately evaluating this chemokine-mediated chemotaxis *in vivo* strictly requires a spontaneous LNM model of ICC, which was technically very challenging to establish. Establishing such *in vivo* models and elucidating the precise downstream mechanisms of the CCR7 pathway in LNM therefore remain important tasks for future investigations.

## Conclusions

SMYD2 expression was observed in metastatic LNs derived from ICC. SMYD2 may regulate CCR7-dependent chemotaxis through SMYD2-phospho-p65^S536^-CCR7 pathway rather than EMT, and associated with poor prognosis via LNM.

## Supplementary Information

Below is the link to the electronic supplementary material.

Supplementary Material 1

Supplementary Material 2

Supplementary Material 3

## Data Availability

Data is provided within the manuscript or supplementary information files.

## Abbreviations

CA19-9: carbohydrate antigen 19-9
CCR7: C-C motif chemokine receptor 7
DFS: Disease free survival
E-cadherin: Epithelial cadherin
EGFP: Enhanced green fluorescent protein
EMT: Epithelial-mesenchymal transition
FBS: Fetal bovine serum
ICC: Intrahepatic cholangiocellular carcinoma
IRS: Immunoreactive scoring
LN: Lymph node
LNM: Lymph node metastasis
N-cadherin: Neural cadherin
NF-κB: Nuclear factor-kappa B
OS: Overall survival
SMYD2: SET and MYND domain-containing 2
siRNA: Small interfering RNA
