# Supplementary material for: SMYD2 regulates lymph node metastasis derived from intrahepatic cholangiocellular carcinoma through CCR7-dependent chemotaxis
Source: Sci Rep. 2026 Jun 1;16:24827. doi: 10.1038/s41598-026-55155-y (PMC13458550; doi:10.1038/s41598-026-55155-y)
Supplement: Supplementary file 1 [file 41598_2026_55155_MOESM1_ESM.docx]

Supplementary Table S1. siRNA sequence.

| **siRNA Name** | **siRNA sequence** | |
| --- | --- | --- |
| siEGFP | Sense | 5’ GCAGCACGACUUCUUCAAG 3’ |
|  | Antisense | 5’ CUUGAAGAAGUCGUGCUGC 3’ |
| siSMYD2 #1 | Sense | 5’ GAUUUGAUUCAGAGUGACA 3’ |
|  | Antisense | 5’ UGUCACUCUGAAUCAAAUC 3’ |
| siSMYD2 #2 | Sense | 5’ GAAUGACCGGUUAAGAGA 3’ |
|  | Antisense | 5’ UCUCUUAACCGGUCAUUUC 3’ |
