# Supplementary material for: SMYD2 regulates lymph node metastasis derived from intrahepatic cholangiocellular carcinoma through CCR7-dependent chemotaxis
Source: Sci Rep. 2026 Jun 1;16:24827. doi: 10.1038/s41598-026-55155-y (PMC13458550; doi:10.1038/s41598-026-55155-y)
Supplement: Supplementary file 2 [file 41598_2026_55155_MOESM2_ESM.docx]

Supplementary Table S2. Primer sequences for qRT-PCR.

| **Gene** | **Primer sequence** | |
| --- | --- | --- |
| GAPDH | Forward | 5’ GCAAATTCCATGGCACCGTC 3’ |
|  | Reverse | 5’ TCGCCCCACTTGATTTTGG 3’ |
| SMYD2 | Forward | 5’ ATCTCCTGTACCCAACGGAAG 3’ |
|  | Reverse | 5’ CACCTTGGCCTTATCCTTGTCC 3’ |
