# Supplementary material for: SMYD2 regulates lymph node metastasis derived from intrahepatic cholangiocellular carcinoma through CCR7-dependent chemotaxis
Source: Sci Rep. 2026 Jun 1;16:24827. doi: 10.1038/s41598-026-55155-y (PMC13458550; doi:10.1038/s41598-026-55155-y)
Supplement: Supplementary file 3 [file 41598_2026_55155_MOESM3_ESM.docx]

(A) **SMYD2**

(B) **E-cadherin**

(C) **N-cadherin**

(D) **β-actin**

**Supplementary Figure S1. Western blotting related to EMT using HuCCT-1.**

(A) **SMYD2**

(B) **E-cadherin**

(C) **N-cadherin**

(D) **β-actin**

**Supplementary Figure S2. Western blotting related to EMT using HuH28.**

(A) **SMYD2**

(B) **p65**

(C) **phosphor-p65^S536^**

(D) **CCR7**

(E) **β-actin**

**Supplementary Figure S3. Western blotting related to SMYD2-phospho-p65^S536^ -CCR7 pathway using HuCCT-1.**
